# Cytogenetic and taxonomic studies of some legless mealybugs (Homoptera, Coccinea, Pseudococcidae)

**DOI:** 10.3897/CompCytogen.v10i4.10503

**Published:** 2016-11-04

**Authors:** Ilya A. Gavrilov-Zimin

**Affiliations:** 1 Zoological Institute, Russian Academy of Sciences, Universitetskaya nab. 1, St. Petersburg, 199034, Russia

**Keywords:** Antonina, Chaetococcus, Komodesia, morphology, chromosomes, new species

## Abstract

A new monotypic genus and species, *Komodesia circuliplurima* **gen. et sp. n.**, from Flores Is. (Indonesia) and the new species, *Antonina diversiglandulosa* **sp. n.**, from Southern Thailand are described and illustrated. Chromosomes of these species and also the ones of *Antonina purpurea* Signoret, 1872 and *Antonina thaiensis* Takahashi, 1942 are studied for the first time: 2n = 30, 20, 12 and 22+Bs correspondingly; the male embryos of all four species demonstrate Lecanoid paternal heterochromatinization of one haploid set of chromosomes. The karyotypes of three widely distributed species, *Antonina pretiosa* Ferris, 1953, *Antonina graminis* (Maskell, 1897) and *Chaetococcus bambusae* (Maskell, 1893), are studied based on material from other regions in comparison with previously published data. Photographs of the karyotypes are provided for the first time for all seven species. The terminological problems connected with the identification and naming of the three scale insect genetic systems, Lecanoid, Comstockioid and Diaspidoid, are discussed.

## Introduction

The world fauna of mealybugs includes at least 26 genera characterized by a strong reduction or a total loss of the legs. These genera were recently combined in the informal generic morphological group *Antonina* Signoret, 1875 (Gavrilov-Zimin 2015). The species of the group share the following characters: loss or strong reduction of the legs, strong reduction of antennae, total or partial sclerotization of the body in mature females, presence of groups of microducts or irregular discoidal pores posteriorly to hind spiracles, absence of ostioles or presence of only poorly developed hind pair of ostioles, loss (with only several exclusions) of circuli, a loss of cerarii (excluding only 1-2 posterior pairs in two monotypic genera), a complete obligate ovoviviparity, a life under the leaf sheath of bamboo grasses, more rarely of other Poaceae grasses or very rarely on some other plants.

Eight of these 26 genera are considered by most modern specialists to be closely related and placed in the separate tribe Serrolecaniini Shinji, 1935. The diagnostic characters of this tribe are caudally directed vulva and groups of microtubular ducts located on ventral cuticle in place of reduced legs or on the surface of peculiar bag-like structures (probably modified hind coxae). The other genera of legless mealybugs perhaps not closely related to each other as well as to Serrolecaniini and demonstrate convergent similarity (see more detailed discussion in Hendricks and Kosztarab 1999). In particular, Serrolecaniini do not include the largest and most widely distributed genus of legless mealybugs – *Antonina*; it includes about 30 species, known mainly from tropical and subtropical regions of the world, especially from Oriental region.

In the present paper a new monotypic genus and species from the Flores Is. (Indonesia), *Komodesia circuliplurima* gen. et sp. n., which must be undoubtedly placed in the tribe Serrolecaniini, and one new species of the genus *Antonina* from Southern Thailand, *Antonina diversiglandulosa* sp. n., are described and illustrated.

**Figure 1.**
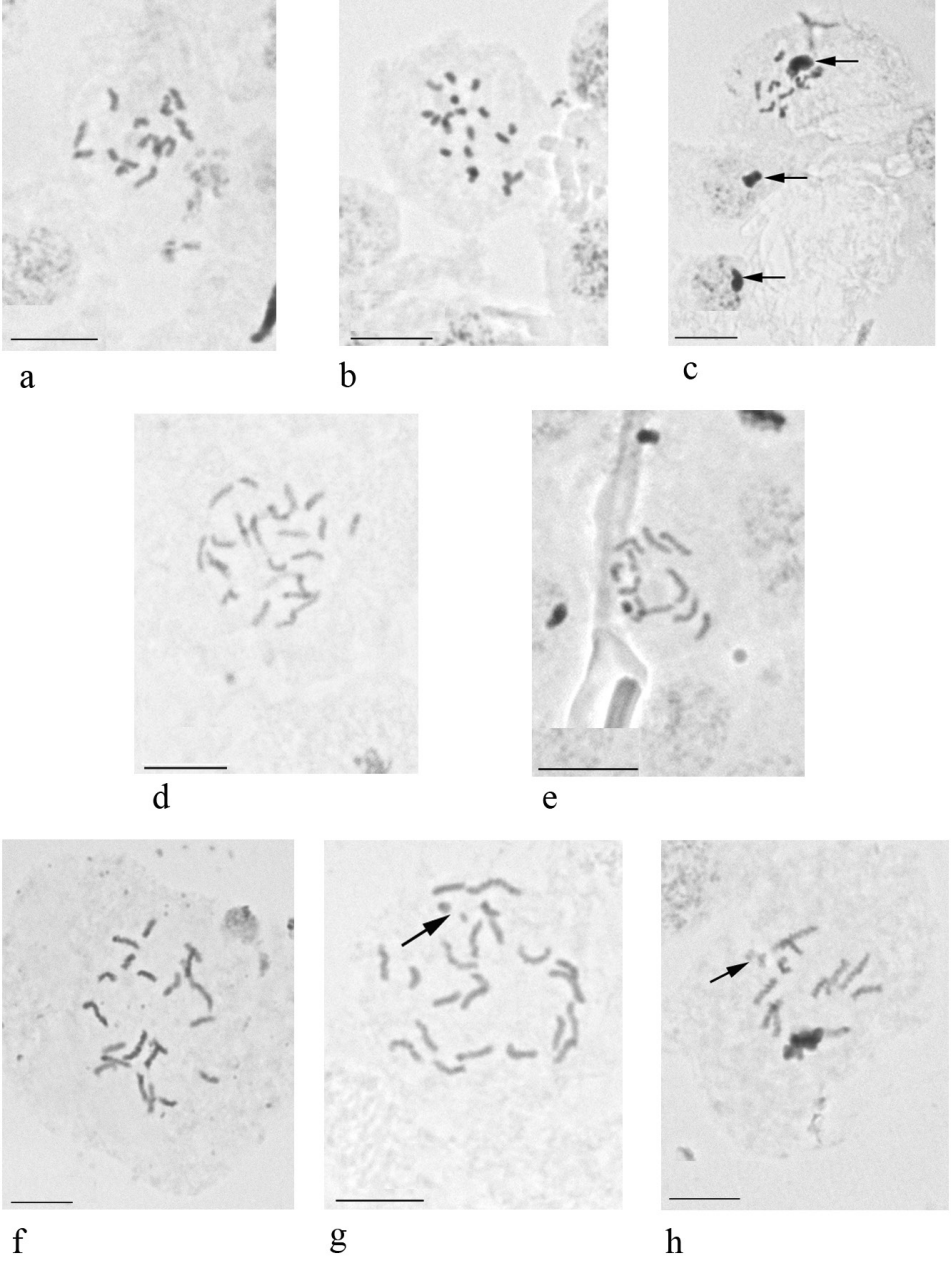
Embryonic cells and chromosomes of species of the genus *Antonina*. **a** *Antonina graminis*, 2n = 16 **b–c** *Antonina diversiglandulosa* sp. n. **b** karyotype, 2n = 20 **c** cells of male embryo with heterochromatinization of paternal set of chromosomes [arrows]) **d** *Antonina pretiosa*, 2n = 24 **e** *Antonina purpurea*, 2n = 12 **f–h** *Antonina thaiensis* (**f** karyotype, 2n = 22 **g** karyotype with 2 B chromosomes [arrow] **h** B chromosomes in male embryo). Bar: 10 µm.

Chromosomal data on legless mealybugs are very scanty. Diploid numbers of chromosomes were previously reported for only 4 species of the genus *Antonina* and one species of *Chaetococcus* Maskell, 1898 (Nur et al. 1987, Gavrilov 2007, Gavrilov and Trapeznikova 2007), but the photo of karyotype was provided only for *Antonina evelynae* Gavrilov, 2003 (Gavrilov 2007). As most of other Pseudococcidae, the legless mealybugs demonstrate the so-called Lecanoid genetic system with specific paternal heterochromatinization of one haploid set of chromosomes in males (Nur 1980, Gavrilov 2007, Gavrilov-Zimin et al. 2015). This heterochromatinization is visible in male tissues during all stages of the life cycle, including embryonal cells (Figs 1c, 8b). The occurrence of heterochromatinization allows detection of the presence of males in the studied populations even when the males and male larvae are not found during the collection of the material. Males of all scale insects are always significantly smaller than females, often live outside of female colonies (even on other host plants or on other parts of the plants) and live as an active imago for a short time only, from several hours to several days. Up to now the males of mealybugs have been collected for some species only, mainly for well-known agricultural pests or for widely distributed species. For example, amongst about 500 species of Palaearctic mealybugs, males were found for no more than 30 species, i.e. about 6% of the faunistic diversity (Danzig and Gavrilov-Zimin 2014). In some species the male embryos (with heterochromatinization) are not detected at all and in these cases the obligate thelytoky is presumed; amongst legless mealybugs two such thelytokous species are known till now, *Antonina graminis* (Maskell, 1897) and *Chaetococcus bambusae* (Maskell, 1893) (Nur et al. 1987).

In the current study the karyotypes for both the new species and for two previously unstudied species of the genus *Antonina*, including the type species of the genus, *Antonina purpurea* Signoret, 1872, were investigated for the first time. Additionally, the earlier noted chromosome numbers of two other widely distributed *Antonina* species and of the type species of *Chaetococcus*, *Chaetococcus bambusae* (McKenzie 1967, Parida and Moharana 1982, Nur et al. 1987), are confirmed based on material collected in other regions, and the photos of the karyotypes are here published for the first time.

## Material and methods

The studied material was collected by the author in different years in Southern Thailand, Indonesia (Flores, New Guinea), Malaysia (Borneo), South France and Morocco. The detailed collecting data are provided below for each species. The numbers with “K” mean unique collecting and preserving numbers for both acetic-ethanol material and Canada balsam slides. All material is deposited at the Zoological Institute, Russian Academy of Sciences (ZIN RAS), St. Petersburg, Russia.

The method of preparation of the morphological Canada balsam slides and method of squashing of the embryonic cells in lactoaceticorcein for chromosomal studies are reported, for example, in Danzig and Gavrilov-Zimin (2014).

## Results and discussion

### Genus *Antonina* Signoret, 1875

#### Antonina graminis

Taxon classificationAnimaliaHemipteraPseudococcidae

(Maskell, 1897)

##### Material.

K 1109, Morocco, 10 km South of Ouarzazate, oasis Fint, under leaf sheathes of undetermined Poaceae grass, 28.IX.2013, Ilya Gavrilov-Zimin.

##### Cytogenetic data.

The chromosome number (2n = 16) of this widely distributed species and its parthenogenetic reproduction were reported for the first time in the book of McKenzie (1967) as unpublished data of “Brown, Beardsley, DeLotto, and Nur” without any other details. Many years later in the review of the chromosome numbers of mealybugs (Nur et al. 1987) these data were repeated with the designation of the collecting country “Jamaica”. Moreover, Parida and Moharana (1982) reported the same chromosome number based on material from India, but without designation of the mode of reproduction. Unfortunately all the above mentioned publications did not include the karyotype photos. During the current study the gravid females of *Antonina graminis* from Morocco (above mentioned locality), from Indonesian part of New Guinea (vicinity of Manokwari) and Malaysian Borneo (Sabah, vicinity of Kinabalu National park) have been examined. In all these series the male embryos (with heterochromatinization) were not detected, i.e. the reproduction in the studied populations was obligate thelytokous. It worth mentioning that the females do have a spermatheca attached to the base of the unpaired oviduct, but without sperm bundles inside. The counting of the chromosomes appeared possible only in the material from Morocco, 2n = 16 (Fig. 1a).

#### Antonina diversiglandulosa sp. n.

Taxon classificationAnimaliaHemipteraPseudococcidae

http://zoobank.org/B6345E97-0876-4503-BD82-1F9A2170BD63

Figs 2 , 3

##### Material.

Holotype, K 1168, Southern Thailand, about 2 km E of Ranong city, under the leaf sheathes of bamboo, 26.XI.2013, Ilya Gavrilov-Zimin, female in a black circle. Paratypes: female on the same slide and 7 females on other slides, all with the same collecting data.

##### Description.

Female. Body broadly oval, up to 3 mm long, sclerotized in mature females (especially posterior segment of abdomen), totally enclosed in thin wax sac. Antennae 2-segmented. Legs totally absent. Anal apparatus located inside long anal tube; anal ring with 6 long setae, each longer than anal tube. Both pairs of ostioles absent. Circulus absent. Multilocular pores of two sizes: larger pores (about 10 µm in diameter) forming band along body margin except several posterior segments; smaller pores (about 6 µm in diameter) forming transverse rows on posterior abdominal sternites IV-VII. Trilocular pores (about 3 µm in diameter) and simple discoidal pores (about 2 µm in diameter) numerous and scattered all over the body surface; trilocular pores which are located inside spiracle atrium are slightly smaller than other trilocular pores. Discoidal pores of irregular structure and size (5-10 µm in diameter) forming two symmetrical groups behind posterior spiracles on abdominal sternites II-VII. Tubular ducts slightly variable in length (being more or less similar with diameter of large multilocular pores), scattered over both body sides. Short flagellate setae sparsely scattered on all segments of body.

Males and morphology of larvae unknown.

**Figure 2.**
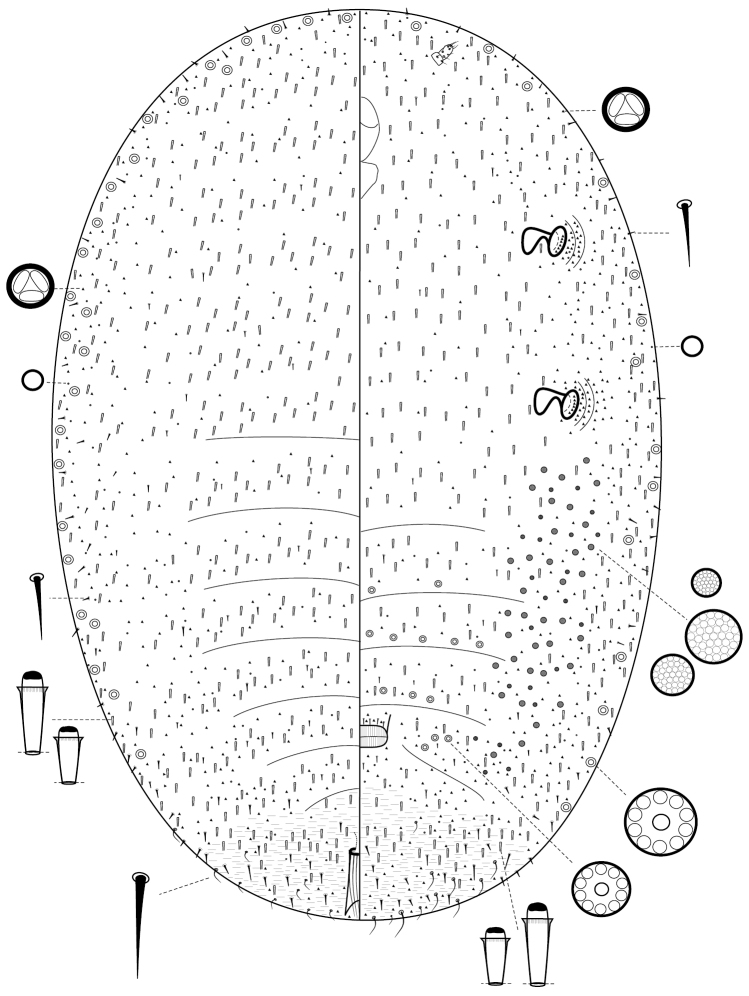
*Antonina diversiglandulosa* sp. n., holotype.

**Figure 3.**
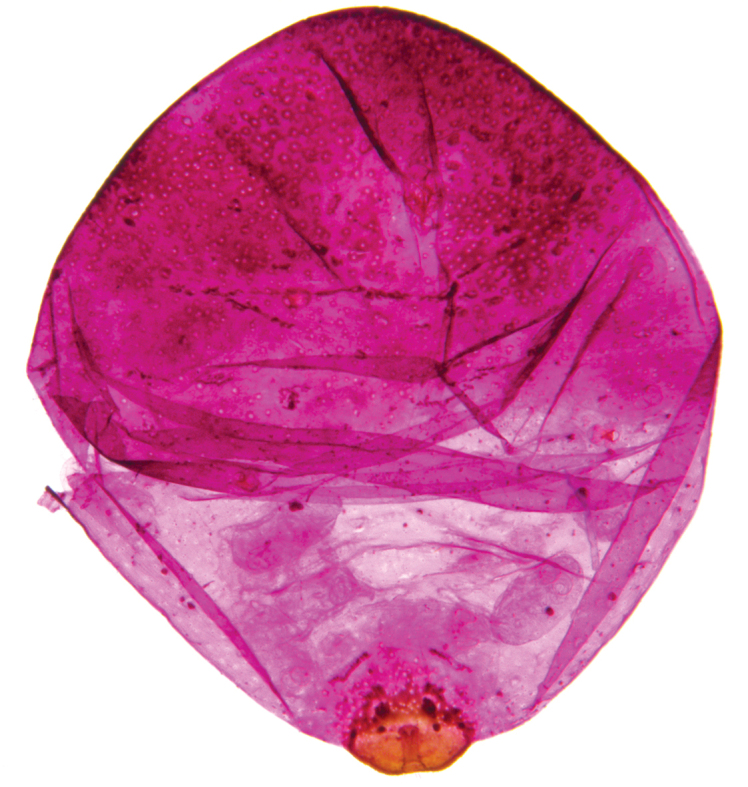
*Antonina diversiglandulosa* sp. n., paratype, prepared mature ovoviviparous female with several fully developed larvae inside the body.

##### Comments.

The Oriental species of *Antonina* were recently revised by Williams (2004) who provided a good identification key. The new species is similar to *Antonina vietnamensis* Williams, 2004 that also has multilocular pores of two sizes (in contrast to all other Oriental species). However, *Antonina diversiglandulosa* sp. n. differs in having many more large groups of irregular pores located behind spiracles on abdominal sternites II-VII (in contrast to compact groups in *Antonina vietnamensis* located on sternites II and III only) and in the absence of circulus. In the neighboring Palaearctic fauna, two-sized multilocular pores are known in *Antonina vera* Borchsenius, 1956 only (Danzig and Gavrilov-Zimin 2015), but this species differs from *Antonina diversiglandulosa* sp. n. in the total absence of irregular pores.

##### Etymology.

The new species name is derived from two Latin words: “diversus” and “glandula”.

##### Cytogenetic data.

Lecanoid heterochromatinization; 2n = 20 (Fig. 1b–c).

#### Antonina pretiosa

Taxon classificationAnimaliaHemipteraPseudococcidae

Ferris, 1953

##### Material.

K 881, Indonesia, New Guinea (Irian Jaya), vicinity of Jayapura city, slopes of Cyclop mountains above Entrop, under the leaf sheathes of bamboo, 1.XI. 2011, Ilya Gavrilov-Zimin.

##### Cytogenetic data.

Nur et al. (1987) studied this species in California (USA) and reported 2n = 24+Bs and sexual reproduction (i.e. with heterochromatinization). Our study of gravid females from New Guinea highlighted 24 chromosomes in embryonic cells (Fig. 1d) and Lecanoid heterochromatinization in the male embryos.

#### Antonina purpurea

Taxon classificationAnimaliaHemipteraPseudococcidae

Signoret, 1872

##### Material.

K 1205, France, Alpes-de-Haute-Provence, Moustiers-Sainte-Marie, on underground stems of undetermined Poaceae grass (probably *Agropyron* sp.), 1.V.2014, Ilya Gavrilov-Zimin.

##### Cytogenetic data.

Lecanoid heterochromatinization; 2n = 12 (Fig. 1e).

#### Antonina thaiensis

Taxon classificationAnimaliaHemipteraPseudococcidae

Takahashi, 1942

##### Material.

K 1167, Southern Thailand, about 2 km E of Ranong city, under the leaf sheathes of bamboo, 26.XI.2013, Ilya Gavrilov-Zimin.

##### Cytogenetic data.

Lecanoid heterochromatinization; 2n = 22, 22+Bs (Fig. 1f–h). Cleavage cells of some embryos show 2 additional B-chromosomes which are also visible in the euchromatic haploid set in males (Fig. 1h).

### Genus *Chaetococcus* Maskell, 1898

#### Chaetococcus bambusae

Taxon classificationAnimaliaHemipteraPseudococcidae

(Maskell, 1893)

##### Material.

K 1172, South Thailand, Phang Nga Province, vicinity of Khura Buri Greenview Resort, under the leaf sheathes of bamboo, 29.XI.2013, Ilya Gavrilov-Zimin.

##### Cytogenetic data.

**Figure 4.**
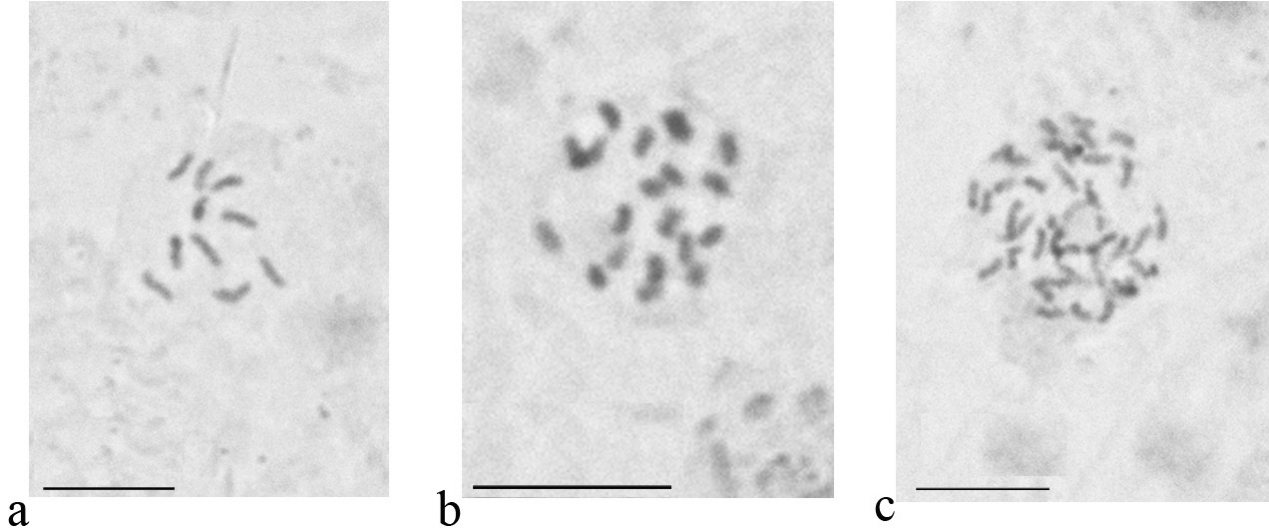
Embryonic cells and chromosomes of *Chaetococcus bambusae*. **a** 2n = 10 **b** 4n = 20 **c** 8n = 40. Bar: 10 µm.

Nur et al. (1987) reported 2n = 10 and parthenogenetic reproduction in this species, based on material from Jamaica. I have studied two populations of this species from Southern Thailand (mentioned above) and from Indonesian New Guinea (vicinity of Jayapura) and also was not able to find male embryos. So, the species is clearly thelytokous. As in the other here studied thelytokous species *Antonina graminis*, the *Chaetococcus bambusae* females have a spermatheca at the base of unpaired oviduct, but without sperm bundles inside. The counting of the chromosomes was possible in the material from Thailand only, 2n = 10 (Fig. 4a). Also, a lot of embryonal cells (probably cells of bacteriome) were with 20 and with 40 chromosomes (Fig. 4b–c).

### Genus Komodesia gen. n.

Taxon classificationAnimaliaHemipteraPseudococcidae

http://zoobank.org/203E81CE-A5AE-4234-A695-DC68E311EBB1

#### Type species.

*Komodesia circuliplurima* sp. n.

#### Description.

Female. Body elongate oval, sclerotized in mature adult females. Three posterior abdominal segments with lateral lobes. Antennae 2-segmented. Legs totally absent. Clypeolabral shield with anterior projection. Vulva caudally directed. Anal apparatus located inside a short anal tube. Both pairs of ostioles absent. Circuli 5 in number, small, round, all of about the same size. Multilocular pores absent. Trilocular pores present. Simple discoidal pores sparsely scattered over all body surface. Microtubular ducts (“duct like pores”) very short, button shaped, forming two symmetrical groups on venter from posterior spiracles to abdominal sternite VI. Tubular ducts (all with small collars) present. Dorsal and ventral surface of body covered by minute flagellate setae.

#### Comments.

The new monotypic genus differs from all other genera of the tribe Serrolecaniini (and all legless mealybugs) in the presence of very short, button-shaped microtubular ducts in groups behind posterior spiracles and in numerous circuli (5 in number).

#### Etymology.

**Figure 5.**
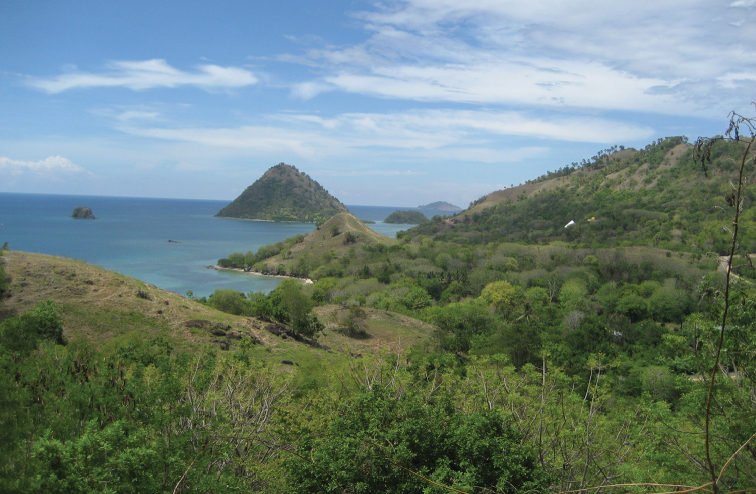
Indonesia, Flores Is., vicinity of Labuan Bajo, the type locality of *Komodesia circuliplurima* gen. et sp. n.

The generic name is after the name of Komodo Island (and appropriate National Park) in vicinity of which the new genus was collected (Fig. 5). Gender feminine.

### Komodesia circuliplurima sp. n.

Taxon classificationAnimaliaHemipteraPseudococcidae

http://zoobank.org/510F70E0-7CBF-4554-BD55-A7FF643AE72C

Figs 6 , 7

#### Material.

Holotype, female, K 979, Indonesia, Flores Is., vicinity of Labuan Bajo, under the leaf sheathes of Poaceae grass, 15.XII.2012, Ilya Gavrilov-Zimin, weakly sclerotized female on the slide. Paratypes: heavily sclerotized female on the same slide, 2 other females on other slide with the same collecting number and data; 2 females K 993, Indonesia, Flores Is., vicinity of Labuan Bajo, Wae Cicu, under the leaf sheath of Poaceae grass, 18.XII.2012, Ilya Gavrilov-Zimin.

#### Description.

Female. Body elongate oval, up to 5 mm long, sclerotized in mature adult females. Three posterior abdominal segments with lateral lobes. Antennae 2-segmented. Legs totally absent. Clypeolabral shield with anterior projection. Vulva caudally directed. Anal apparatus located inside short anal tube; clear structure of anal ring invisible in available females, but the ring bears 6 long setae, which longer than anal tube. Both pairs of ostioles absent. Circuli 5 in number, small, round, all of about the same size. Multilocular pores absent. Trilocular pores (each about 4 µm in diameter) scattered over dorsal surface of cephalothorax and anterior abdominal tergites, forming sparse groups around spiracles on venter and totally absent on four posterior abdominal segments. Simple discoidal pores (each about 2 µm in diameter) sparsely scattered on all body surface. Microtubular ducts very short (each about 3 µm in diameter), button-shaped, forming two symmetric groups on venter from posterior spiracles to abdominal sternite VI. Tubular ducts (all with small collars) of three main sizes: largest ducts about two times wider than diameter of trilocular pore numerous on head; mid-sized ducts similar in width to trilocular pore, forming marginal band along all body venter; smallest tubular ducts about 1.5 times thinner than diameter of trilocular pore, scattered on abdominal tergites and numerous in medial and submedial zones of venter. Dorsal and ventral surface of body covered with rare minute flagellate setae; significantly longer setae present on both sides of two posterior abdominal segments.

Males and morphology of larvae unknown.

**Figure 6.**
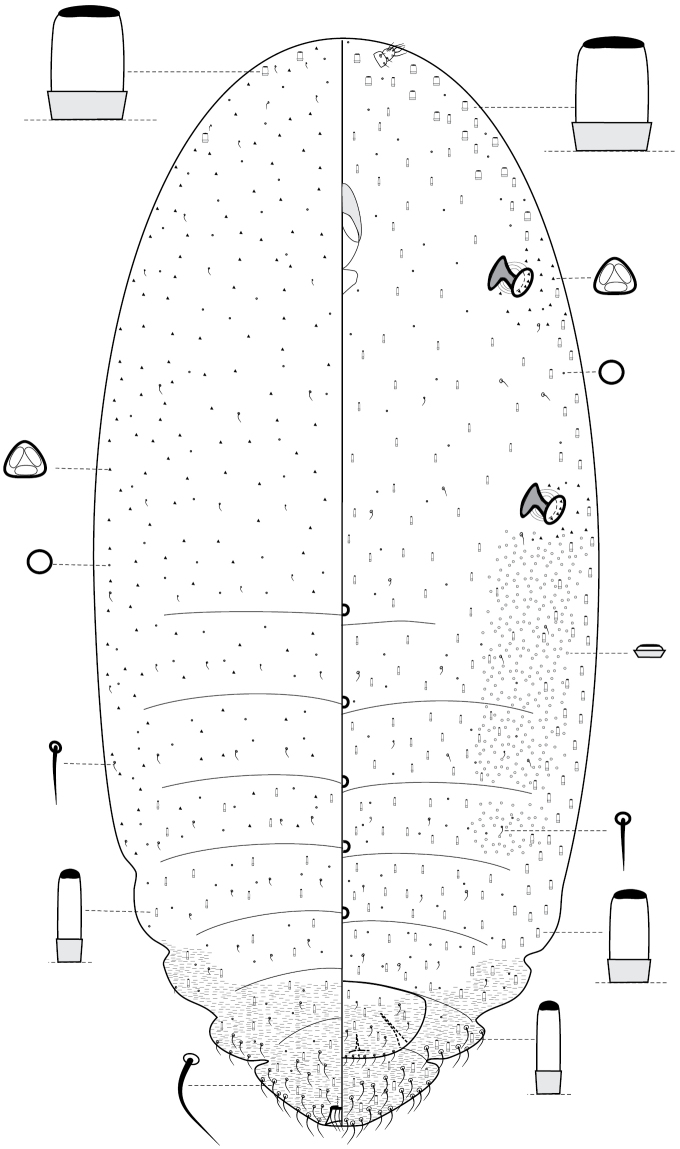
*Komodesia circuliplurima* gen. et sp. n., holotype.

**Figure 7.**
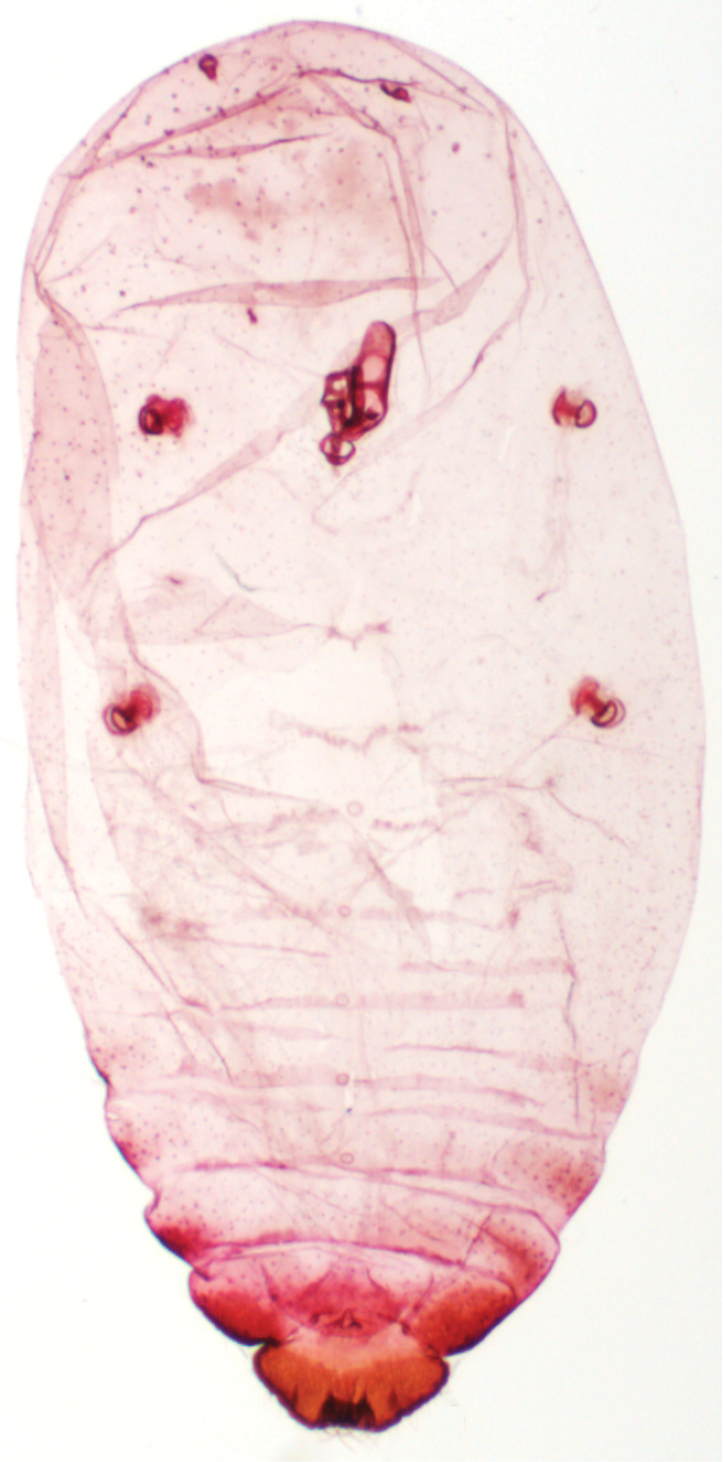
*Komodesia circuliplurima* gen. et sp. n., holotype, prepared female on slide.

#### Etymology.

The new species name is derived from two Latin words: “*circulus*” meaning circle and “*plurimus*” meaning many in plural and means: “with many circuli”.

#### Cytogenetic data.

**Figure 8.**
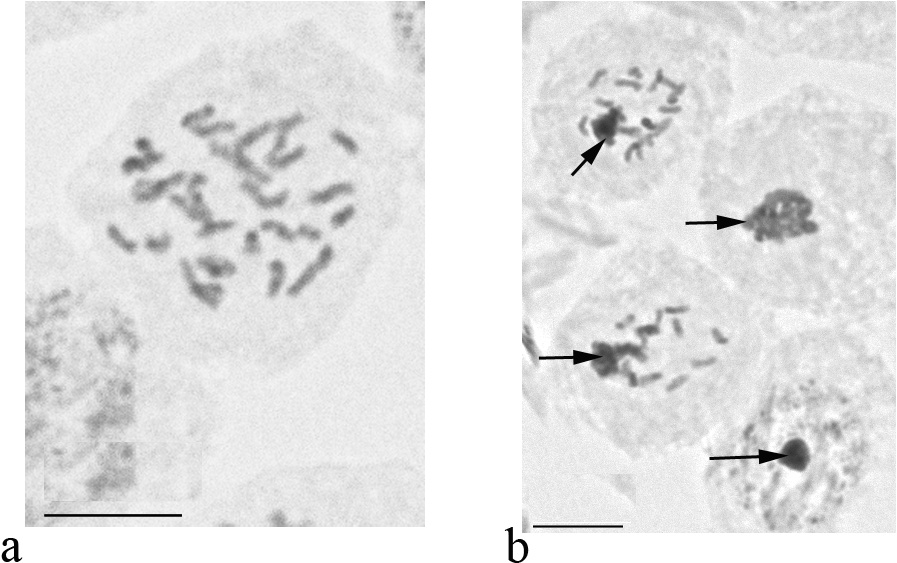
Embryonic cells and chromosomes of *Komodesia circuliplurima* gen. et sp. n. **a** 2n = 30 **b** cells of male embryo with heterochromatinization of paternal set of chromosomes (arrows). Bar: 10 µm.

Lecanoid heterochromatinization; 2n = 30 (Fig. 8). *Komodesia circuliplurima* gen. et sp. n. demonstrates the highest number of chromosomes currently known in legless mealybugs.

### Discussion of cytogenetic data

Together with the data reported in this paper, the chromosome numbers are now known in nine species of legless mealybugs from 3 genera (Table 1).

**Table 1.** Chromosomal numbers and genetic systems of legless mealybugs (S – sexual reproduction, P – parthenogenesis without detailing, T – thelytoky, L – Lecanoid heterochromatinization in embryos, Bs – B-chromosomes).

Species	2n	Genetic system	Reference
*Antonina crawi* Cockerell, 1900	12	S	Nur et al. 1987 [Hawaii, USA]
*Antonina evelynae* Gavrilov, 2003	12	L	Gavrilov 2007 [Sochi, Russia]
*Antonina graminis* (Maskell, 1897)	16 16 16	P ? T	Nur et al. 1987 [Jamaica] Parida and Moharana 1982 [India] Present study [Morocco]
*Antonina diversiglandulosa* sp. n.	20	L	Present study [Thailand]
*Antonina pretiosa* Ferris, 1953	24+ Bs 24	S L	Nur et al. 1987 [California, USA] Present study [New Guinea, Indonesia]
*Antonina purpurea* Signoret, 1872	12	L	Present study [France]
*Antonina thaiensis* Takahashi, 1942	22+Bs	L	Present study [Thailand]
*Chaetococcus bambusae* (Maskell,1893)	10 10	P T	Nur et al. 1987 [Jamaica] Present study [Thailand]
*Komodesia circuliplurima* gen. et sp. n.	30	L	Present study [Flores Is., Indonesia]

In spite of a small number of studied species, six different diploid chromosome numbers are known at present and it seems that legless mealybugs are more diverse in this character than other groups of mealybugs (see the information for all Pseudococcidae in the catalogue of Gavrilov 2007). It correlates with morphological diversity of this group, most genera of which are monotypic and distinctly differ from each other. It is also interesting that the modal number of chromosomes of all mealybugs (2n = 10) is known as of now for only one species of legless mealybugs (*Chaetococcus bambusae*), that may be considered as an additional evidence of intensive evolution in the group.

Most of studied species (7 out of 9) of legless mealybugs possesses the Lecanoid genetic system of reproduction. Traditionally, in the works of old American cytogeneticists (Hughes-Shrader 1948, Brown 1965, Nur 1980 and others) three different genetic systems (Lecanoid, Comstockioid [Comstockiella] and Diaspidoid [Diaspidid]) with heterochromatinization and obligate bisexual reproduction were considered. In the special terminological paper (Gavrilov and Kuznetsova 2007) we suggested unifying the endings of these names and use them not only for bisexual reproduction (as earlier), but also for facultative arrhenotoky and deuterotoky when males also have the same heterochromatinization (see our argumentation in the cited paper). However, at that time we did not discuss another terminological problem: the differences between the Lecanoid system and its derivate Comstockioid system are very difficult to detect in practice being visible only just prior to prophase I of spermatogenesis; moreover, some species demonstrate Lecanoid and Comstockioid systems (and different variants of Comstockioid!) in the same male (Nur 1980). In practice, it leads to the situation when it is impossible to clearly identify the genetic system in absolute majority of karyotyped species, because the male larvae (with meiotic and premeiotic cells inside) are collected very rarely and even if collected some of them only may provide a good material for chromosome studies. Consequently, most species listed by Nur et al. (1987) are noted as having “sexual reproduction”, without exact designation of the genetic system. We propose that only two systems with heterochromatinization, Lecanoid and Diaspidoid which show discrete differences from each other at all stages of the life cycle, should be accepted. On the other hand, the term “Comstockioid” is proposed to use only for the derivative variant of spermatogenesis within the Lecanoid system. Thus, for the species of legless mealybugs studied in the present paper I designate the Lecanoid system based only on the presence of paternal genome heterochromatinization in the embryos.

## Supplementary Material

XML Treatment for Antonina graminis

XML Treatment for Antonina diversiglandulosa

XML Treatment for Antonina pretiosa

XML Treatment for Antonina purpurea

XML Treatment for Antonina thaiensis

XML Treatment for Chaetococcus bambusae

XML Treatment for Komodesia

XML Treatment for Komodesia circuliplurima
